# The role of endothelial-to-mesenchymal transition in cancer progression

**DOI:** 10.1038/sj.bjc.6604662

**Published:** 2008-09-16

**Authors:** S Potenta, E Zeisberg, R Kalluri

**Affiliations:** 1 Division of Matrix Biology, Department of Medicine, Beth Israel Deaconess Medical Center and Harvard Medical School, Boston, MA, USA; 2 Department of Cell Biology, Harvard Medical School, Boston, MA, USA; 3 Department of Biological Chemistry and Molecular Pharmacology, Harvard Medical School, Boston, MA, USA; 4 Harvard-MIT Division of Health Sciences and Technology, Boston, MA, USA

**Keywords:** endothelial-to-mesenchymal transition, EndMT, cancer-associated fibroblasts, angiogenesis, endothelium, fibrosis

## Abstract

Recent evidence has demonstrated that endothelial-to-mesenchymal transition (EndMT) may have a significant role in a number of diseases. Although EndMT has been previously studied as a critical process in heart development, it is now clear that EndMT can also occur postnatally in various pathologic settings, including cancer and cardiac fibrosis. During EndMT, resident endothelial cells delaminate from an organised cell layer and acquire a mesenchymal phenotype characterised by loss of cell–cell junctions, loss of endothelial markers, gain of mesenchymal markers, and acquisition of invasive and migratory properties. Endothelial-to-mesenchymal transition -derived cells are believed to function as fibroblasts in damaged tissue, and may therefore have an important role in tissue remodelling and fibrosis. In tumours, EndMT is an important source of cancer-associated fibroblasts (CAFs), which are known to facilitate tumour progression in several ways. These new findings suggest that targeting EndMT may be a novel therapeutic strategy, which is broadly applicable not only to cancer but also to various other disease states.

As an integral component of the circulatory system, the endothelium can be defined as the single-cell layer of mostly squamous epithelium that provides the inner cell lining of blood vessels and lymphatics (Junqueira and Carneiro, 2005). Endothelial cells can exhibit a wide range of phenotypic variability depending on local physiologic needs throughout the vascular tree (Chi *et al*, 2003). Furthermore, in pathologic states, the endothelium can be affected in a number of ways; perhaps the most remarkable is an extreme form of endothelial plasticity known as endothelial-to-mesenchymal transition (EndMT).

During EndMT, resident endothelial cells delaminate from an organised cell layer and invade the underlying tissue (Figure 1). This so-called mesenchymal phenotype can be characterised by loss of cell–cell junctions, acquisition of invasive and migratory properties, loss of endothelial markers, such as CD31 (also known as platelet endothelial cell adhesion molecule-1 (PECAM-1)), and gain of mesenchymal markers, such as fibroblast-specific protein 1 (FSP1; also known as S100A4) or *α*-smooth muscle actin (*α*SMA; Potts and Runyan, 1989; Nakajima *et al*, 2000; Armstrong and Bischoff, 2004; Arciniegas *et al*, 2007; Zeisberg *et al*, 2007a, 2007b). Previous studies of EndMT have focused largely on embryonic development of the heart. However, recent evidence suggests that EndMT can occur postnatally in a variety of pathologic settings, including cancer and cardiac fibrosis (Zeisberg *et al*, 2007a, 2007b). There is also growing evidence that EndMT may be associated with select types of endothelium in the body.

With regard to cancer, EndMT accounts for up to 40% of cancer-associated fibroblasts (CAFs). Cancer-associated fibroblasts play an important role in tumour progression and can alter the microenvironment in several ways. In particular, CAFs deposit various extracellular matrix molecules and secrete paracrine factors that can directly affect the behaviour of many different cell types within the tumour. Furthermore, CAFs release potentially oncogenic signals, such as transforming growth factor-*β* (TGF-*β*), and are a principle source of host-derived vascular endothelial growth factor (VEGF), which promotes angiogenesis (Kalluri and Zeisberg, 2006). Here, we highlight the recent findings of EndMT as a source of CAFs, with a discussion of proposed mechanisms and therapeutic implications.

## Endothelial-to-mesenchymal transition *vs* epithelial-to-mesenchymal transition

Endothelial-to-mesenchymal transition is often categorised as a specialised form of epithelial-to-mesenchymal transition (EMT). Epithelial-to-mesenchymal transition can occur in many epithelial cell types and is a critical process in embryogenesis (Thiery and Sleeman, 2006). In the setting of disease, EMT has been demonstrated during epithelial injury and can also occur in individual tumour cells as an important mechanism of invasion and metastasis (Batlle *et al*, 2000; Cano *et al*, 2000; Zavadil and Bottinger, 2005; Thiery and Sleeman, 2006; Tse and Kalluri, 2007).

Epithelial-to-mesenchymal transition has been extensively studied and has provided a useful framework for guiding research on EndMT. Both EMT and EndMT give rise to cells that have a similar mesenchymal phenotype, and current evidence suggests that both utilise common signalling pathways. However, further studies are needed to validate this notion, as there exist some key differences between endothelial cells and other types of epithelial cells. In particular, endothelial cells express distinct cell–cell junctional proteins, different cytoskeletal proteins, different signalling machinery, and different surface markers (Table 1). The importance of these differences as they relate to EndMT needs to be fully investigated.

## Endothelial-to-mesenchymal transition in development

Endothelial-to-mesenchymal transition was first observed in developmental studies of heart formation (Markwald *et al*, 1975, 1977). In this context, a subset of endothelial cells lining the primitive heart tube are triggered to acquire a mesenchymal phenotype and invade the surrounding tissue, where they subsequently participate in forming the valves and septa of the adult heart (Armstrong and Bischoff, 2004). So far, studies of embryonic heart formation have provided the majority of current knowledge about EndMT. Mechanistic studies have demonstrated a role for TGF-*β*, bone morphogenic protein (BMP), and Notch pathways (Potts and Runyan, 1989; Nakajima *et al*, 2000; Armstrong and Bischoff, 2004; Timmerman *et al*, 2004; Thiery and Sleeman, 2006).

## Endothelial-to-mesenchymal transition in cancer and angiogenesis

**Figure 2:**
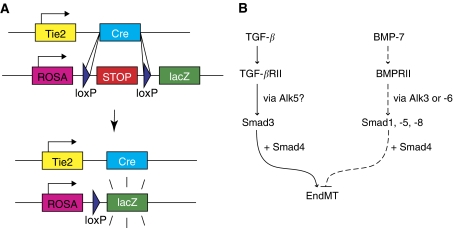
Endothelial-to-mesenchymal transition in cancer and cardiac fibrosis. (**A**) The Tie2-Cre;ROSA-STOP-lacZ reporter mouse is an important strain for tracking cells of endothelial origin during EndMT. In this mouse, Cre expression is driven by the Tie2 promoter, which is known to be active in endothelial cells. The Cre recombinase acts by permanently excising genomic DNA regions that are flanked by loxP sites (floxed). In this case, Tie2-driven Cre activity removes a floxed stop cassette, thereby allowing lacZ expression to be driven by the constitutive ROSA26R promoter (ROSA) without the need for continued Tie2 activity. (**B**) During cardiac fibrosis, TGF-*β* signalling promotes EndMT through Smad3 transcriptional activity. In endothelial cells, TGF-*β* is known to activate Alk5, which then activates Smad3. However, the role of Alk5 has not been explicitly demonstrated during EndMT in cardiac fibrosis. EndMT was also shown to be inhibited by rhBMP-7 (dashed lines). BMP-7 is known to act through a different set of Smads, namely Smad1, -5, and -8. However, the precise mechanisms whereby BMP-7 inhibits EndMT are not yet known.

Recently, studies have demonstrated that EndMT can occur in a variety of pathologic states including cancer (Zeisberg *et al*, 2007a) and cardiac fibrosis (Zeisberg *et al*, 2007b). With regard to cancer, EndMT is now recognised as a unique source of CAFs (Zeisberg *et al*, 2007a). Cancer-associated fibroblasts are known to facilitate tumour progression in several ways (reviewed by Kalluri and Zeisberg, 2006), and are a key component of tumour stroma. The discovery of EndMT in tumours was reported in a recent study that investigated two different mouse models of cancer and demonstrated that a substantial proportion of CAFs arise through EndMT. These CAFs were identified as a unique population of cells that coexpress the endothelial marker CD31 along with one of the mesenchymal markers, FSP1, or *α*SMA. Approximately, 40% of FSP1+ CAFs were also found to be CD31+, as were 11% of *α*SMA+ CAFs. Furthermore, this study also investigated tumours grown in *Tie2-Cre;R26R-lox-STOP-lox-lacZ* transgenic mice, a reporter strain that allows all cells of endothelial origin to be irreversibly labelled with *lacZ* expression (Figure 2A). Similar results were found: among FSP1+ CAFs, 30% were also lacZ+, and among *α*SMA+ CAFs, 12% were lacZ+. These data suggest that EndMT is an important mechanism for CAF recruitment to the tumour stroma and that these CAFs may have a unique role in tumour progression. Coincidentally, TGF-*β* signalling is a known mediator of EndMT (Nakajima *et al*, 2000) and is abundantly expressed in many different tumours (Zeisberg *et al*, 2007a), therefore suggesting that EndMT may be mediated by TGF-*β* signalling in this context. Nevertheless, the molecular mechanism of EndMT in tumours has not yet been specifically studied, but is likely to involve similar pathways as described in the setting of cardiac fibrosis (Figure 2B).

Taken together, these results demonstrated that up to 40% of CAFs might be derived through EndMT. This study has furthermore demonstrated that angiogenic vessels can undergo EndMT. We speculate that EndMT may play a role in angiogenic sprouting by enabling the so-called tip cells, which lead an emerging vascular plexus, to migrate into adjacent tissue. As migratory cells with no lumen (Gerhardt *et al*, 2003), tip cells have a phenotype that appears to be consistent with EndMT. At the angiogenic front, these migrating endothelial cells are exposed to growth factors and interstitial matrix molecules, such as type I collagen, which differ from their normal vascular basement membrane components (Davis and Senger, 2005). Perhaps in response to these factors, some endothelial cells may undergo EndMT and maintain their mesenchymal phenotype indefinitely. Moreover, previous reports have suggested that vascular support cells, such as pericytes and/or smooth muscle cells, may arise from the endothelium itself, and therefore EndMT may be an important mechanism in recruiting such mural cells during angiogenesis (Armulik *et al*, 2005). Furthermore, these vascular support cells are an important component of mature vessels (Armulik *et al*, 2005), and therefore EndMT may play an important role in stabilizing the neovasculature during vasculogenesis and angiogenesis. This would be consistent with the finding that a subpopulation of EndMT-derived cells express *α*SMA, a well-established marker for pericytes and vascular smooth muscle cells (Armulik *et al*, 2005).

## Endothelial-to-mesenchymal transition in cardiac fibrosis and other diseases

Another recent study has further validated the notion that EndMT can occur postnatally in the context of disease. This study addressed EndMT during cardiac fibrosis, a common feature of most forms of heart failure (Zeisberg *et al*, 2007b). In fact, postnatal EndMT has been most extensively studied in this setting of cardiac fibrosis. Here, approximately 27–35% of all fibroblasts in fibrotic heart tissue were found to arise through EndMT. Furthermore, this study demonstrated a role for Smad3-dependent TGF-*β* signalling during EndMT *in vivo* (Figure 2B). Interestingly, mice treated with recombinant human BMP-7 (rhBMP-7, another member of the TGF-*β* superfamily known to antagonise the effects of TGF-*β*) exhibited a significant reduction both in fibrosis as well as EndMT (Figure 2B).

In addition to the studies described above, there is evidence to suggest that EndMT may occur in many other disease settings, such as chronic pulmonary hypertension (Zhu *et al*, 2006; Arciniegas *et al*, 2007), atherosclerosis (Mironov *et al*, 1995), wound healing (Sarkisov *et al*, 1988; Lee and Kay, 2006), and in both acute and chronic kidney injury (Zeisberg *et al*, 2008). These emerging reports have characterised EndMT primarily in terms of marker expression, but have not addressed the precise molecular mechanisms of EndMT in disease. More importantly, the functional role of EndMT in each of these scenarios has not yet been determined.

## Signalling during EndMT

In addition to the *in vivo* studies described above, a number of reports have also demonstrated the induction of EndMT *in vitro*. To date, many different endothelial cell types, including both human and mouse, have demonstrated EndMT when exposed to TGF-*β* or Notch ligands *in vitro* (Frid *et al*, 2002; Ishisaki *et al*, 2003; Noseda *et al*, 2004; Timmerman *et al*, 2004; Zeisberg *et al*, 2007a, 2007b). These studies have led to a greater understanding of the mechanisms driving EndMT. *In vivo* studies have further substantiated that EndMT can be modulated in response to manipulations of the TGF-*β* or Notch pathways (Sanford *et al*, 1997; Timmerman *et al*, 2004; Zeisberg *et al*, 2007b). Still, it is not clear whether Notch, TGF-*β*, or a combination of both pathways provides the initiating signal under physiologic conditions *in vivo*. It is also likely that other signalling pathways interact with TGF-*β* and Notch to mediate EndMT. For example, VEGF, NFAT, BMP, Wnt/*β*-catenin, ErbB, and NF1/Ras have been implicated in EndMT during cardiac development (Armstrong and Bischoff, 2004), but have yet to be specifically explored in the context of pathology. In fact, the majority of mechanistic work related to EndMT has been performed in the context of embryonic development, and therefore may not reflect the same mechanisms that occur in disease.

Downstream of these signalling events, the transcriptional networks mediating EndMT also remain largely unidentified. In one series of experiments, cells that undergo EndMT exhibited an increased expression of the *Snail* family of transcriptional repressors (Carmona *et al*, 2000; Romano and Runyan, 2000; Timmerman *et al*, 2004). Snail proteins are also known to be upregulated during EMT, where they play a critical role in disrupting cell–cell junctions (Batlle *et al*, 2000; Cano *et al*, 2000; Nieto, 2002). In the context of EndMT, *Snail* repressors are believed to downregulate VE-cadherin, thereby disrupting adherens junctions and allowing endothelial cells to delaminate and undergo EndMT. It is unknown whether other effectors of EMT, such as Twist (Thiery and Sleeman, 2006), CArG box-binding factor A (CBF-A), and KRAB-associated protein 1 (KAP-1) (Venkov *et al*, 2007), are also involved in EndMT.

## Perspectives and therapeutic implications

Studies of EndMT have revealed a novel mechanism of fibroblast and mural cell recruitment that is likely to be involved in many different disease settings. Fibroblasts are known to have an important role in tissue remodelling and fibrosis (Tomasek *et al*, 2002; Kalluri and Zeisberg, 2006; Zeisberg *et al*, 2007a, 2007b), although previously very little was known about the origin of fibroblasts in damaged tissues. Various mechanisms have been proposed, including the activation of local fibroblasts within the affected tissue, recruitment of bone marrow-derived precursors, and EMT occurring in nearby epithelia (Iwano *et al*, 2002). However, in addition to these mechanisms, it is now clear that EndMT accounts for a considerable proportion of these fibroblasts, estimated at 27–35% during cardiac fibrosis and up to 40% in tumours (Zeisberg *et al*, 2007a, 2007b). This suggests that fibroblasts can be recruited from a combination of sources, although the relative contribution from each source may vary in different disease states. It is also possible that certain vascular beds are more likely to be affected by EndMT. The endothelium is highly heterogenous and dynamic by nature, and therefore future studies will need to address EndMT in the context of this inherent variation in endothelial phenotypes. For instance, angiogenic vessels in tumours seem to be particularly prone to EndMT.

Perhaps most importantly, the recent discoveries of EndMT in different diseases suggest that modulating EndMT may represent a promising new treatment modality. The endothelium itself is an attractive target for drug delivery because it lies in direct contact with the bloodstream. We hypothesise that therapies directed at inhibiting EndMT may delay tumour progression, perhaps as a result of impaired angiogenesis or CAF recruitment. Possible treatment strategies may target the TGF-*β* and/or BMP signalling pathways. The mouse studies described above have demonstrated that systemic administration of rhBMP-7 significantly reduced EndMT during cardiac fibrosis (Zeisberg *et al*, 2007b). Follow-up studies should be pursued to address possible effects of BMP-7 treatment on EndMT in tumours and to identify other EndMT targets.

Furthermore, inhibiting EndMT may be broadly applicable to various disease states. For example, preventing EndMT during chronic organ fibrosis may significantly delay disease progression and allow patients to maintain adequate organ function for a longer period of time. Nevertheless, additional studies are needed to identify the precise molecular mechanisms of EndMT in disease and to determine which signalling components might be viable therapeutic targets. A promising place to start may be to further examine the developmental defects in EndMT. Just as normal developmental mechanisms are often recapitulated in certain disease states (Reya *et al*, 2001), the corresponding developmental defects might provide unique insights into possible treatments for those diseases. In conclusion, the study of EndMT represents an exciting new frontier in vascular biology that will continue to provide novel insights into the mechanisms of human disease.

## Figures and Tables

**Figure 1:**
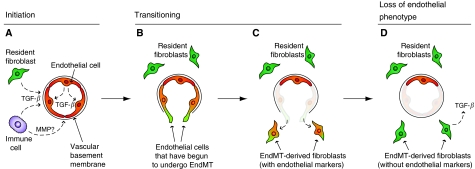
Stages of EndMT. (**A**) Endothelial-to-mesenchymal transition may be initiated by autocrine and/or paracrine inflammatory signals originating from within the surrounding tissue, such as TGF-*β*. Possible sources include resident fibroblasts (green) or immune cells (purple). Alternatively, the endothelium (red) may undergo EndMT in direct response to vascular injury. The vascular basement membrane is likely to be degraded by matrix metalloproteinases (MMPs) derived from local immune cells. (**B**–**C**) Transitioning endothelial cells (red/green) acquire a migratory phenotype, invade under the vascular basement membrane, and begin to express mesenchymal markers, such as FSP1, while still expressing endothelial markers. (**D**) Cells that have undergone EndMT (green) have lost their endothelial phenotype. These EndMT-derived cells contribute to the local fibroblast population and are likely to produce various growth factors, such as TGF-*β*. It is not yet known whether the affected vessels are repopulated, and if they remain functional after resident endothelial cells have departed.

**Table 1:** Comparison of epithelial, endothelial, and mesenchymal cells

	**Epithelial cell**	**Endothelial cell**	**Mesenchymal cell**
*Cell–cell junctions:*	adherens junctions w/E-cadherin tight junctions desmosomes	adherens junctions w/VE-cadherin *limited* tight junctions	None (or focal)
			
*Organised cell layer:*	✓	✓	No
			
*Apico-basolateral polarity:*	✓	✓	No
			
*Basement membrane:*	✓	✓	No, but makes interstitial matrix
			
*Migratory:*	No	No	✓
			
*Intermediate filament:*	Cytokeratin	Vimentin	Vimentin
			
*Markers:*	E-cadherin, claudins, occludins, desmoplakin, cytokeratin, mucin-1	CD31, VE-cadherin, Tie1, Tie2, VEGFR	FSP1, *α*SMA, vimentin, fibronectin, vitronectin, collagen types I and III
